# Antifungal Activity of Maytenin and Pristimerin

**DOI:** 10.1155/2012/340787

**Published:** 2012-05-22

**Authors:** Fernanda P. Gullo, Janaina C. O. Sardi, Vânia A. F. F. M. Santos, Fernanda Sangalli-Leite, Nayla S. Pitangui, Suélen A. Rossi, Ana C. A. de Paula e Silva, Luciana A. Soares, Julhiany F. Silva, Haroldo C. Oliveira, Maysa Furlan, Dulce H. S. Silva, Vanderlan S. Bolzani, Maria José S. Mendes-Giannini, Ana Marisa Fusco-Almeida

**Affiliations:** ^1^Laboratory of Clinical Mycology, Department of Clinical Analysis, Faculty of Pharmaceutical Sciences, UNESP, Rua Expedicionários do Brasil 1621, 14801-902 Araraquara, SP, Brazil; ^2^Institute of Chemistry, UNESP, Rua Professor Francisco Degni 55, 14800-900 Araraquara, SP, Brazil

## Abstract

Fungal infections in humans have increased alarmingly in recent years, particularly in immunocompromised individuals. Among the infections systemic candidiasis, aspergillosis, cryptococcosis, paracoccidioidomycosis, and histoplasmosis mortality are more prevalent and more severe in humans. The current high incidence of dermatophytosis is in humans, especially as the main etiologic agents *Trichophyton rubrum* and *Trichophyton mentagrophytes*. Molecules pristimerin and maytenin obtained from the plant *Maytenus ilicifolia* (Celastraceae) are known to show various pharmacological activities. This study aimed to evaluate the spectrum of antifungal activity of maytenin and pristimerin and their cytotoxicity in human keratinocytes (NOK cells of the oral mucosa). It was concluded that the best spectrum of antifungal activity has been shown to maytenin with MIC varying from 0.12 to 125 mg/L, although it is also active with pristimerin MIC ranging between 0.12 and 250 mg/L. Regarding the toxicity, both showed to have high IC_50_. The SI showed high pristimerin against some species of fungi, but SI maytenin was above 1.0 for all fungi tested, showing a selective action of fungi. However, when comparing the two substances, maytenin also showed better results. The two molecules can be a possible prototype with a broad spectrum of action for the development of new antifungal agents.

## 1. Introduction

Fungal infections are mainly caused by opportunistic fungi and are usually associated with immunosuppression [1]. Over the past two decades, invasive fungal infections have emerged as a major threat to immunocompromised patients, since species of *Aspergillus*, *Candida*, *Cryptococcus,* and *Histoplasma *emerging pathogens such as *Fusarium* and *Trichophyton* can cause infection when defenses of host are broken [1]. Paracoccidioidomycosis is a fungal infection that is very important, which affects a large percentage of the rural population of Latin America [2].

There is now a great interest in the discovery of new molecules of natural origin for the treatment of various diseases [3]. Natural products have provided a wide variety of drugs and have become an alternative to large demand for new antifungal drugs [4]. In Brazil, the use of medicinal plants in traditional medicine has increased considerably in recent years. The wide distribution of natural resources in Brazilian ecosystems and natural diversity of chemical components and provides the country with potential bioactive materials [5].

**Figure 1:**
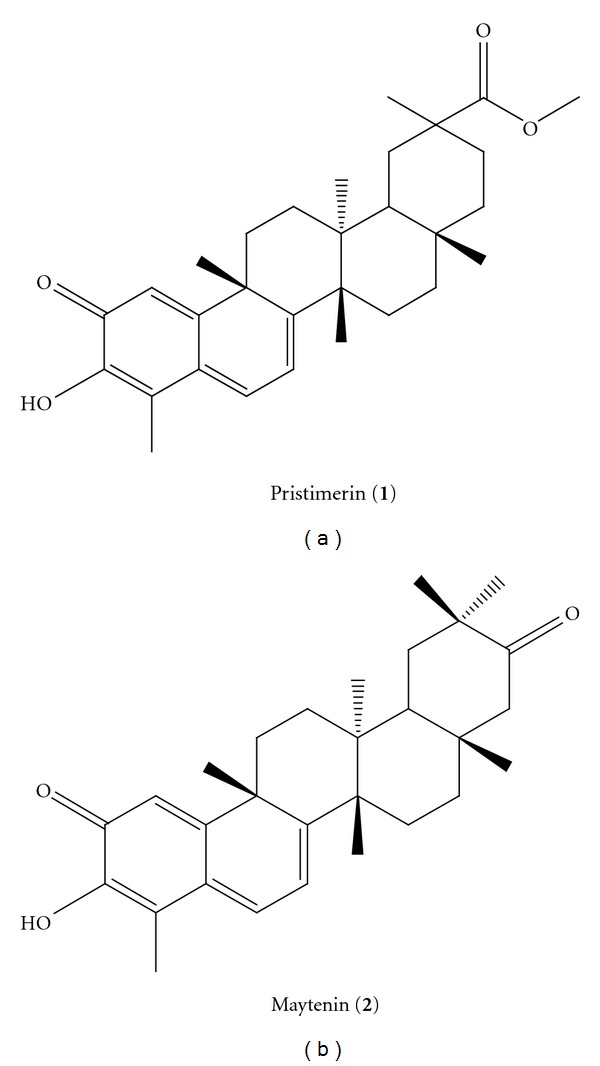
Structures of the isolated quinonemethide triterpenes from *M. ilicifolia*, pristimerin (****1****) and maytenin (**2**).

*Maytenus ilicifolia* (Celastraceae), popularly known as “*espinheira santa*,” has been used in traditional medicine since the mid-1920s [6]. The secondary metabolites, maytenin and pristimerin (Figure 1), are classified as quinonemethide triterpenoids and mainly isolated from the bark of the roots of mature *M. ilicifolia* plants [7]. Several studies have shown that maytenin exhibits strong antimicrobial activity against Gram-positive and Gram-negative organisms, but there are no studies detailing the effects of these substances on fungi [8].

 The need to discover new antifungal molecules and natural products is of great importance for the development of new therapeutic tools. This paper proposes the study of the potential antifungal potential of maytenin and pristimerin against fungi, an agent of important mycoses. The potential broad spectrum of these molecules was evaluated, and the cytotoxicity of these substances in cell lines was evaluated, suggesting possible prototypes of a broad spectrum of components for the treatment of mycoses.

## 2. Methods

### 2.1. Microorganisms

 ATCC strains and clinical isolates belong to the mycology collection of the Laboratory of Clinical Mycology, Department of Clinical Analysis, Faculty of Pharmaceutical Sciences, UNESP, Araraquara, were used in the current study. The strains used were: *Candida albicans *ATCC 90028, *Candida krusei *ATCC 6258, *Candida parapsilosis *ATCC 22019, *Candida glabrata *ATCC 90030, *Candida tropicalis *ATCC 750, *Cryptococcus neoformans* var. *grubii *ATCC 90012, *Histoplasma capsulatum* (G217B), *Aspergillus niger* ATCC 16404, *Aspergillus fumigatus* ATCC 7100, *Trichophyton interdigitalis* ATCC 40131. Clinical isolates from *Paracoccidioides brasiliensis *(18), *Trichophyton rubrum* and *Trichophyton mentagrophytes *(102), *Cryptococcus neoformans* var. *grubii* resistant to fluconazole (R30), two clinical isolates from *Cryptococcus neoformans* var. *grubii* susceptible to fluconazole (S26 and S27), and *Cryptococcus gattii* (118) isolate from animals, resistant to fluconazole, and *Histoplasma capsulatum* (M238P) (lung bat) were also used.

### 2.2. Minimum Inhibitory Concentration (MIC)

 All microorganisms were tested for susceptibility to specific commercial drugs for the treatment of each gender. The test for yeast was carried out in accordance with the microdilution method described according to the M27-S3 of the CLSI (Clinical and Laboratory Standards Institute) (2008), with modifications. The filamentous fungi susceptibility testing was performed according to the M38-A2 of the CLSI (2008) [9] with modifications to determine the minimum inhibitory concentration (MIC).

Two pure substances extracted from *Maytenus ilicifolia*, maytenin and pristimerin, were prepared as described by Scorzoni et al. 2007 [10]. The antifungal drugs were diluted according to CLSI M27-S3 [9]. Inoculums were prepared in RPMI-1640 (Sigma-Aldrich, St. Louis, MO, USA) with L-glutamine, without sodium bicarbonate, supplemented with 2% glucose, and buffered to a pH of 7.0 using 0.165 M MOPS, (Sigma-Aldrich, St. Louis, MO, USA). The yeast suspension was adjusted to a final concentration of 1.0 × 10^4^ CFU/mL in RPMI-1640 and for filamentous fungi, a suspension of microconidia was adjusted to 2.5 to 5.0 × 10^3^. In the 96-well plates, substances were added in serial dilutions, starting from a concentration of 250 mg/L to 0.48 mg/L. The plates were incubated in a shaker at 37°C/150 rpm to a specific time determined for each microorganism. The reading of MIC was performed by spectrophotometry at 490 nm and confirmed using Alamar Blue (Sigma-Aldrich, St. Louis, MO, USA).

### 2.3. Minimum Fungicide Concentration (MFC)

 A qualitative analysis of fungal viability was performed, by transferring a portion of the wells to a plate with Sabouraud (Sigma-Aldrich, St. Louis, MO, USA) medium and incubated at 37°C during the time determined for each fungal agent. The MFC was determined as the lowest concentration of the extract that did not allow the growth of any fungal colony on the solid medium after the incubation period [11]. A visual reading was performed to confirm the death or growth inhibition provided by the antifungal substances, maytenin and pristimerin.

### 2.4. Cell Cytotoxicity Assay

 The cytotoxicity of maytenin and pristimerin was assessed by MTT for cell lines NOK (keratinocyte oral mucosa), obtained from the American Type Culture Collection (Manassas, VA, USA). The cells were grown in own culture medium NOK (Keratinocytes—SFM, GIBCO), and maintained at 36.5°C. A concentration ranging from 2.5 to 5.0 × 10^4^ cells/mL was used for the formation of monolayer cells. The concentrations of pure substances were kept in contact with the cells for 24 hours. After the incubation period, the cells treated with the MTT reagent has an additional 5 mg/mL added, (Sigma-Aldrich, St. Louis, MO, USA) and the cells were incubated again for another 4 h. After the formation of formazan crystals, 100 *μ*L of isopropanol was added to solubilize the precipitate and allow the reading of the result by changing the color of the medium [12]. The absorbance of formazan was quantified using an ELISA reader (enzyme-linked immunosorbent assay) set at 560 nm. As a positive control test, hydrogen peroxide was used.

## 3. Results

### 3.1. Minimum Inhibitory Concentration (MIC)

Table 1 present the results of the antifungal activity of maytenin and pristimerin, isolated from *Maytenus ilicifolia*. Maytenin activity was classified as potent for both yeasts and for filamentous fungi, when compared to positive controls itraconazole and amphotericin B; since the MIC ranged between 0.12 and 62.5 mg/L. Pristimerin previously showed a significant variation in the action potential. For yeasts, the pristimerin showed potent activity, with the exception of *C. glabrata* ATCC 90030 which showed moderate activity and *C. albicans* ATCC 90028, which presented low antifungal activity. For the filamentous fungi, pristimerin showed moderate activity, with the exception of *Trichophyton mentagrophytes* ATCC 40131, which showed a MIC of 62.5 mg/L and was classified as having potent activity.

### 3.2. Minimum Fungicide Concentration (MFC)

 Minimum fungicide concentration was performed to confirm cell death in MIC through observation of no colony growth in a rich medium. The maytenin showed fungicide activity for most yeasts. No growth of *C. albicans* colonies was observed, and the death of yeast occurred in a higher dilution concentration (Table 1). For the filamentous fungi, MFC was confirmed by testing cell viability, which occurs in MIC fungal cell death (Table 1).

 Pristimerin presented a greater difference between the MIC and MFC. Moreover, the difference between the MIC and the value of qualitative analysis of fungal viability was greater than the difference presented by maytenin (Table 1).

 For the fungus *Paracoccidioides brasiliensis* and the filamentous fungi *Aspergillus niger *and* fumigatus*, the qualitative test of fungal viability was flawed, since the growth of colonies cannot be observed on a solid medium.

### 3.3. Cell Cytotoxicity Assay

 The cytotoxicity assay for the NOK cell line showed more than 80% of cell viability in MIC maytenin concentrations. The substance pristimerin showed cell viability above 80% in normal cells (Table 1).

## 4. Discussion

Plants have been used in medicine for a long period of time, since they are easy to obtain and apply various diseases [4, 13]. Regarding the search for new antifungal agents, the ideal must have a broad spectrum of fungicidal activity without causing toxicity to the host [14]. The treatment of fungal infections is not always effective because of resistance to drugs in addition to presenting high toxicity for human cells. For this reason, there is a continuing search for new drugs which are more potent antifungal, but safer, than existing drugs [15].

The present study showed that pristimerin and maytenin had potente action on the fungi studied (Table 1), but maytenin showed the best results. The exception was *Histoplasma capsulatum *isolated M238P and *Paracoccidioides brasiliensis* which showed MIC equal for both substances.

Alanís-Garza et al., 2007 [16] studied the anti-*Histoplasma* activity of extracts obtained from various plants and showed MICs between 16 and 125 mg/L. This study showed an MIC for ATCC strains of *H. capsulatum* 0.97 and less than 0.48 mg/L pristimerin and maytenin respectively, while for clinical isolate, the MIC for the two substances was less than a 0.48 mg/L. Compared with few literary data of natural products active against this fungal, pristimerin and maytenin can be considered prototypes that are excellent anti-*Histoplasma*.

Few studies have searched for new drugs from natural products for *P. brasiliensis* [17, 18]. There are reports of the activity of anti-*Paracoccidioides* of *Piper regnellii* and “*Baccharis dracunulifolia*” 30 mg/L [17, 19]. The results expressed in this study were excellent for *P. brasiliensis*, MIC of 0.12 mg/L for both molecules at a concentration 65 times lower than the results of the studies mentioned above.

The systemic mycosis caused by yeasts *Cryptococcus *spp. has increased because of AIDS [20]. In the current results indicate that the molecules had an excellent MIC for the yeasts this genus, ranging from 0.48 to 3.9 mg/L for maytenin and 0.97 to 7.8 for pristimerin. Another study revealed the antifungal activity of extracts of *Maytenus undata* against *C. neoformans*, which showed MIC of 0.09 mg/L after 24 h, and 0.18 mg/L after 48 h [21]. The species *Candida* are classified as the fourth most common pathogen in hospitals and are associated with increased mortality of bloodstream infections due to these fungi having high resistance to existing antifungal [22]. For this yeast, the pristimerin showed potent activity, with the exception of *C. glabrata* ATCC 90030, which showed moderate activity, and *C. albicans* ATCC 90028, which obtained low antifungal activity. Maytenin showed moderate antifungal activity for this specie. For* C. albicans*, maytenin and pristimerin showed fungicide activity; however, the *Candida* non-*albicans* the result was fungistatic, except for *C. glabrata* in contact pristimerin.

 Although dermatophyte infections are restricted to certain areas of the epidermis, they can be invasive and cause serious injury [23]. Due to the high incidence there is a great need to find new drugs which act on the dermatophytes. Maytenin showed potent activity with MIC ranging from 1.95 to 3.9 mg/L. For filamentous fungi, the pristimerin showed moderate activity, with the exception of *Trichophyton mentagrophytes* ATCC 44131, which showed a MIC of 62.5 mg/L. Many studies have demonstrated the plant antifungal potential against these dermatophytes [23–25], as the study of Lau et al. 2010 [24], that evaluated extracts *Eucalypti Folium* and *Fructus Psoraleae Globuli*. Both the pure compounds effectively inhibit the growth of *T. mentagrophytes* and *T. rubrum* [25].

 Invasive aspergillosis is an important cause of mortality in transplant patients [26]. Maytenin had better results against *Aspergillus niger* with an MIC value of 0.97 mg/L, whereas the MIC for *A. fumigatus *was 125 mg/L and pristimerin was similar for both 250 mg/L. Maytenin showed good fungicide activity for most filamentous fungi, while pristimirin showed high MIC (Table 1).

 On the other hand, the cytotoxicity tests performed with NOK (keratinocytes oral mucosa) showed that the experimental substances are not cytotoxic to this cell examined in experiment (Table 1). Although most of the antifungal agents available on the market are of synthetic origin, natural products of the study received the attention of researchers, mainly, due to the occurrence of unwanted factors, such as the resistance of some strains the conventional antifungal agents—especially in immunocompromised individuals—and the presence of these toxic effects. Figueiredo et al. (1998) found antimalarial activity of various substances, including the pristimerin but the cytotoxicity was only for the 17-(methoxycarbonyl)-28-*nor*-isoiguesterin in adenocarcinoma cell line HT-29 [27].

 With respect to selectivity index (SI), it is known that the higher the SI of a substance, the greater is its security. In our study we found that the maytenin substance had SI above 1.0 for all species tested, so we can demonstrate the safe use of this. Likewise, the pristimerin presented high SI against some fungal species, as described for *H. capsulatum* (SI 13.40). However, when comparing the two substances, maytenin still showed better results (Table 1).

## 5. Conclusion

 The results of this study indicate the potential use of maytenin and pristimerin for the treatment of fungal infections, which showed a potent antifungal activity against the fungi studied. Therefore, the data obtained are promising. Although the medicinal plant “*Maytenus ilicifolia*” is consolidated in the treatment of gastritis and ulcers more pharmacological studies will be necessary to evaluate these molecules as antifungal prototypes.

## Figures and Tables

**Table 1:** MIC values and quantitative analysis of fungal cellular viability of pure substances maytenin and pristimerin front of the yeasts and filamentous pathogenic strains causing the most mycoses and evaluation of cytotoxic activity in NOK cells and selectivity index from the ratio of the IC_50_ and MFC.

	Maytenin MIC/MFC	Pristimerin MIC/MFC	Amphotericin B reference values MIC	Itraconazole reference values MIC	Maytenin IC_50_ (mg/L)/SI	Pristimerin IC_50_ (mg/L)/SI
*Candida albicans* ATCC 90028	62.50/62.50	250.00/250.00	0.50–2.00	—	265.7/4.25^♦^	*
*Candida krusei* ATCC 6258	15.62/62.50	7.81/62.50	0.25–2.00	0.12–0.50	78.33/1.25^♦^	24.25/0.38^♦^
*Candida parapsilosis* ATCC 22019	15.62/31.25	125.00/125.00	0.25–1.00	0.06–0.25	78.33/2.50^♦^	*
*Candida tropicalis* ATCC 750	15.62/31.25	31.25/62.52	0.50–2.00	—	78.33/2.50^♦^	203.98/3.26^♦^
*Candida glabrata* ATCC 90030	31.25/31.25	125.00/125.00	0.25–1.00	—	203.98/6.52^♦^	*
*Cryptococcus neoformans* ATCC 90012	0.48/0.48	0.97/0.97	0.50	0.125	2.53/5.29^♦^	3.92/4.04^♦^
*Cryptococcus neoformans* R30	3.90/3.90	7.81/7.81	1.00	>32.00	22.18/5.68^♦^	24.25/3.10^♦^
*Cryptococcus neoformans *isolated 26	0.48/0.48	1.95/1.95	0.50	0.125	2.53/5.29^♦^	6.41/3.28^♦^
*Cryptococcus neoformans *isolated 27	0.48/0.48	3.90/3.90	0.50	0.125	2.53/5.29^♦^	20.10/5.15^♦^
*Cryptococcus gattii* 118	1.95/1.95	3.90/3.90	1.00	>32.00	9.84/5.05^♦^	20.10/5.15^♦^
*Paracoccidioides brasiliensis *Pb18	<0.12/-	<0.12/-	0.015–0.25	<0.0039	**	**
*Histoplasma capsulatum *ATCC 0G217B	0.97/0.97	0.48/0.48	0.06–0.25	0.25–2.00	3.70/3.81^♦^	6.43/13.40^♦^
*Histoplasma capsulatum *isolated M238P	0.48/0.48	0.48/0.48	0.06–0.25	0.25–2.00	2.53/5.29^♦^	6.43/13.40^♦^
*Aspergillus fumigatus *ATCC 7100	125/-	>250/-	0.12–2.0	0.12- > 16.00	*	*
*Aspergillus niger *ATCC 16404	0.97/0.97	250/250	0.12–0.5	0.12–1.00	^♦^3.70/3.81	*
*Trichophyton rubrum *clinical isolated	1.95/1.95	250/250	—	0.03–4.00	^♦^9.84/5.05	*
*Trichophyton mentagrophytes *ATCC 40131	3.9/3.9	62.5/250	—	0.03–0.25	^♦^22.18/5.68	150.24/0.6^♦^
*Trichophyton mentagrophytes *clinical isolated 102	3.9/3.9	125/250	—	0.03–0.25	^♦^22.18/5.68	*

MIC: minimum inhibitory concentration; MFC: minimum fungicide concentration.

^♦^ IC_50_/IS values in NOK cells.

* IC_50_/IS not apply because the MIC and MFC values are above 62.5 mg/L.

** IC_50_/IS not apply because the MIC and MFC values are below 0.12 mg/L.

IC_50_: 50% inhibitory concentration. SI: selectivity index obtained from the relationship between the IC_50_ (NOK) by MFC for each fungi.
